# Using large language models to directly screen electronic databases as an alternative to traditional search strategies such as the Cochrane highly sensitive search for filtering randomized controlled trials in systematic reviews

**DOI:** 10.1017/rsm.2025.10034

**Published:** 2025-10-10

**Authors:** Viet-Thi Tran, Carolina Grana Possamai, Isabelle Boutron, Philippe Ravaud

**Affiliations:** 1 Center for Research in Epidemiology and Statistics (CRESS), Université Paris Cité and Université Sorbonne Paris Nord, INSERM, INRAE, Paris, France; 2 Centre d’Epidemiologie Clinique, AP-HP, Hôpital Hôtel Dieu, Paris, France; 3 Centre Cochrane France, Paris, France; 4 Department of Epidemiology, Mailman School of Public Health, Columbia University, New York, NY, USA

**Keywords:** Large language models, search strategy, systematic reviews

## Abstract

A critical step in systematic reviews involves the definition of a search strategy, with keywords and Boolean logic, to filter electronic databases. We hypothesize that it is possible to screen articles in electronic databases using large language models (LLMs) as an alternative to search equations. To investigate this matter, we compared two methods to identify randomized controlled trials (RCTs) in electronic databases: filtering databases using the Cochrane highly sensitive search and an assessment by an LLM.

We retrieved studies indexed in PubMed with a publication date between September 1 and September 30, 2024 using the sole keyword “diabetes.” We compared the performance of the Cochrane highly sensitive search and the assessment of all titles and abstracts extracted directly from the database by GPT-4o-mini to identify RCTs. Reference standard was the manual screening of retrieved articles by two independent reviewers.

The search retrieved 6377 records, of which 210 (3.5%) were primary reports of RCTs. The Cochrane highly sensitive search filtered 2197 records and missed one RCT (sensitivity 99.5%, 95% CI 97.4% to100%; specificity 67.8%, 95% CI 66.6% to 68.9%). Assessment of all titles and abstracts from the electronic database by GPT filtered 1080 records and included all 210 primary reports of RCTs (sensitivity 100%, 95% CI 98.3% to100%; specificity 85.9%, 95% CI 85.0% to 86.8%).

LLMs can screen all articles in electronic databases to identify RCTs as an alternative to the Cochrane highly sensitive search. This calls for the evaluation of LLMs as an alternative to rigid search strategies.

## Highlights

### What is already known?

A critical step in systematic reviews involves the definition of a search strategy, with keywords and Boolean logic, to filter electronic databases.

### What is new?

We hypothesize that LLMs could directly screen all articles in electronic databases as an alternative to rigid search equations. To investigate this matter, we compared two methods to identify RCTs in electronic databases: the Cochrane highly sensitive search and an assessment by a GPT (OpenAI) of all articles published. The Cochrane highly sensitive search missed one article and had poor specificity. In contrast, assessment of all titles and abstracts extracted from the electronic database by GPT yielded perfect sensibility and high specificity.

### Potential impact for RSM readers

In contrast to current uses of LLMs to accelerate search in systematic reviews by mimicking and improving human tasks, we show that it is possible to envision the process of systematic reviews differently, accounting for the ability of LLMs to treat large volumes of data automatically and at low costs.

## 1 Background

Synthesizing evidence from randomized controlled trials (RCTs) in systematic reviews and meta-analyses is a cornerstone of evidence-based medicine. Yet, current methods for systematic reviews are slow and resource intensive, requiring up to a year for a team when following recommended approaches.^1^^,^ ^2^ The exponential growth of medical literature and the demand for timely evidence synthesis have thus driven the development of automated tools to accelerate the review process, from literature screening to data extraction and risk of bias assessment.^3^

One of the first steps of the review process involves the formulation of a search strategy with specific keywords and Boolean logic to filter electronic databases such as PubMed. Guidance documents for systematic reviews recommend combining broad set of search terms for each concept (e.g., population, intervention), with the “OR” operator within concepts and the “AND” operator between concepts.^4^ To identify studies with the appropriate design, the Cochrane Collaboration recommends adding the validated “highly sensitive search strategy” to identify RCTs in systematic reviews focused on interventional studies.^5^^,^ ^6^ While such approach maximizes sensitivity, it also often results in the retrieval of a large number of irrelevant records, which must then be screened manually.

A recent scoping review of large language model (LLM) applications for evidence synthesis has found that 41% targeted the search strategy by assisting in refining Medical Subject Headings (MeSH) terms, formulating PubMed queries, or translating queries across databases.^3^ In contrast to these approaches, we hypothesize that LLMs’ ability to treat large volumes of data automatically could be used to completely bypass the search strategy step by directly screening all articles in electronic databases as an alternative to rigid search equations.^7^

## 2 Objectives

To investigate this matter, we compared the performance of two strategies to identify RCTs in a sample of studies extracted from electronic databases: (1) the Cochrane highly sensitive search (i.e., a traditional search strategy using keywords and Boolean logic) and (2) the assessment by an LLM (GPT from OpenAI) of all articles in the sample. The reference standard was the double assessment by human reviewers.

## 3 Methods

On January 25, 2025, we retrieved a sample of studies indexed in PubMed (without limiting to MEDLINE records) with a publication date between September 1 and September 30, 2024. As there were 179,000 records indexed in PubMed in the time frame of the study, we used the keyword “Diabetes” to reduce the number of publications included in this study. Search strategies are provided in Supplementary Section 1.

Our objective was to identify primary reports of RCTs, excluding secondary and post-hoc analyses, protocols, and systematic reviews and meta-analyses. We considered as an RCT any prospective study designed to evaluate the causal effect of one or more interventions by randomly allocating eligible participants into two or more groups and comparing outcomes between these groups. This definition excluded, for instance, animal studies in which a disease was randomly induced to observe biomarker differences, as disease induction was not considered an intervention.

We performed the Cochrane highly sensitive search (2008 version) using the equation described in the Cochrane Handbook (Index test 1).^6^ We performed the GPT assessment of all titles and abstracts directly extracted from the database using a zero-shot prompt (i.e., the prompt included no example), inspired by a previous study, and ran on GPT-4o-mini through the application programming interface (API) of OpenAI (Index test 2) (Table 1).^7^ The prompt was initially tested with GPT-4o to ensure high accuracy and then transitioned to GPT-4o-mini to reduce computational costs, as recommended by OpenAI^8^ (Supplementary Section 2). The reference standard was the manual screening of the abstracts of retrieved articles by two independent reviewers (VTT and CG), blinded from the results of the index tests. Abstracts that did not explicitly describe the study design, but whose objectives were compatible with an RCT, were retained as potential primary reports of RCTs. Conflicts between reviewers were resolved through discussion and consensus during regular meetings.

**Table 1:** Prompt and example of output from the GPT model

User prompt	You are an experienced systematic reviewer. You are screening medical research articles based on their title and abstracts to identify reports of randomized controlled trials. Appraise the following title and abstract. Summarize the elements that let you think that this study is (or is not) the report of a randomized controlled trial (including crossover, factorial trials, etc.). You must exclude secondary analyses, systematic reviews, meta-analyses, pooled analyses, and protocols of randomized controlled trials. ### TITLE ### abhishek, f., et al. (2024). 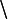 “comparative analysis of bariatric surgery and non-surgical therapies: impact on obesity-related comorbidities. 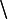 ” cureus 16(9): e69653. ### ABSTRACT ### Obesity is associated with a broad spectrum of comorbidities, including metabolic dysregulation, cardiovascular complications, and socioeconomic impacts. Traditional lifestyle interventions often yield transient results in weight management, while bariatric surgery offers a promising alternative. This systematic review adhered to the Preferred Reporting Items for Systematic Reviews and Meta-Analysis (PRISMA) guidelines and focused on randomized controlled trials comparing bariatric surgery (e.g., Roux-en-Y gastric bypass (RYGB), adjustable gastric banding (AGB), and sleeve gastrectomy (SG)) with non-surgical therapies (drug therapy and lifestyle modifications) in the management of obesity-related comorbidities, particularly hypertension and type 2 diabetes mellitus (T2DM). We comprehensively searched databases like PubMed, PMC, and EBSCO using Medical Subject Headings (MeSH) terms related to obesity, bariatric surgery, and non-surgical treatments. We included seven studies involving participants aged 18–64 published within the last 6 years. We rigorously assessed these studies for quality and extracted data to evaluate outcomes such as weight loss, diabetes remission rates, hypertension management, and renal function. The review found that bariatric surgery consistently resulted in substantial and sustained weight loss compared to non-surgical therapies. Surgical interventions significantly improved hypertension control, reduced cardiovascular risks, and enhanced glycemic control in diabetic patients. The effectiveness of bariatric surgery in promoting diabetes remission was attributed not only to weight loss but also to physiological changes in gastrointestinal anatomy, gut hormones, and energy balance regulation. Limitations identified in the literature included variations in study methodologies, follow-up durations, and patient characteristics, which limited direct comparisons and generalizations. Future research should incorporate more extended follow-up periods and standardized methods to further validate these findings’ durability and broad applicability across diverse patient populations. In conclusion, bariatric surgery emerges as an effective treatment option for managing obesity-related comorbidities, particularly hypertension and T2DM. While acknowledging the inherent risks and complexities associated with surgical interventions, ongoing research and clinical innovations are crucial to optimizing patient outcomes and reducing the global burden of obesity-related diseases.”
LLM	Based on the title and abstract provided, it is clear that this article cannot be classified as a report of a randomized controlled trial (RCT) for several reasons: **Type of Study Described**: The title specifies a “comparative analysis” of bariatric surgery and non-surgical therapies, which suggests it is evaluating existing studies rather than presenting original research. The abstract indicates that the authors conducted a systematic review, which is an analysis of multiple studies rather than a new RCT. **Systematic Review Mentioned**: The abstract explicitly states that the work is a “systematic review.” This indicates that the authors are aggregating results from previously conducted research studies rather than presenting new RCT results. Because systematic reviews provide summaries and insights based on the analysis of RCTs and other study designs, they do not present original data from a single trial. **No Original Research Findings**: The abstract does not report findings from a new trial or present original research data that corresponds to a randomized trial. Instead, it summarizes findings from multiple studies regarding bariatric surgery and its effects. **Focus on Current Literature**: The authors mention including “seven studies,” which are likely existing RCTs that have already been published. This implies they are synthesizing these studies’ results rather than carrying out a new RCT on their own. In summary, the title and abstract clearly indicate that this is a systematic review of already published randomized controlled trials comparing bariatric surgery and non-surgical therapies, and therefore, it does not represent a new randomized controlled trial itself. The absence of original study design, methods, and results typical of RCTs further supports this classification.”
User prompt	Based on the title and abstract and your previous analysis, should the record go to full-text screening (the record should go to full-text screening if it is a report of a randomized controlled trial or if there are not enough elements to decide whether it is one or not)? This is less problematic to move texts to full-text screening than to miss a potential randomized controlled trial.
LLM	Given the information presented in the title and abstract, this record should **not** go to full-text screening. The reasons for this conclusion are as follows: **Clear Indication of a Systematic Review**: The abstract explicitly states that this work is a systematic review. Systematic reviews summarize and analyze existing literature, including randomized controlled trials and other study designs, but they do not present new data from original randomized controlled trials. **Absence of Original Study**: The abstract indicates that the authors have reviewed other studies, specifically mentioning that they included seven studies and provided a summary of their findings, rather than conducting their own trial. **No Elements Suggesting Original Data Collection**: There are no indications in the title or abstract that the authors conducted a randomized controlled trial themselves. Since the work is an analysis of existing literature, it does not meet the criteria for inclusion as a new RCT.
	Thus, since it is definitively identified as a systematic review and does not contain original trial data or an active report of a randomized controlled trial, there is no lack of clarity regarding the study type. Therefore, moving this record to full-text screening would not be necessary, as it is clear from the title and abstract that this is not a randomized controlled trial.
User prompt	Can you summarize your previous answer with simply “yes” or “no”?
LLM	No

*Note*: The prompt leverages the dialogue capability of GPT models to induce reflection on the eligibility of studies and improve performance. The example abstract is that of a systematic review; as it contains keywords specific to RCTs, it is not filtered by the Cochrane highly sensitive search.^10^

We assessed the performance of the two index tests by calculating the sensitivity and specificity with 95% confidence intervals (CI).

## 4 Results

The electronic search retrieved 6377 records, of which 210 (3.5%) were primary reports of RCTs according to the reference standard.

The Cochrane highly sensitive search filtered 2197 records (34.4%), which included 209 primary reports of RCTs, 1988 false positives, and one false negative trial which used the abbreviation “RCT” rather than words such as “random*” in its title and abstract (sensitivity 99.5%, 95% CI 97.4% to100%; specificity 67.8%, 95% CI 66.6% to 68.9%).^9^

Assessment of all titles and abstracts directly extracted from the electronic database by GPT filtered 1080 records (i.e., a reduction of 50.8% of the number of records from the Cochrane highly sensitive search). GPT included all 210 primary reports of RCTs from the reference standard and 870 false positives (sensitivity 100%, 95% CI 98.3% to100%; specificity 85.9%, 95% CI 85.0% to 86.8%).

## 5 Discussion

In this proof-of-concept study, we showed that LLMs could directly screen all articles in PubMed to identify RCTs, as an alternative to filtering databases with the Cochrane highly sensitive search. Use of LLMs reduced the number of articles to manually screen by 50%; enabled the identification of one reference missed by the Cochrane highly sensitive search; and improved the explainability of the search by providing reasons for each study excluded (Table 1).

In this study, we used the keyword “Diabetes” for feasibility reasons as there were 179,000 records indexed in PubMed in the time frame of the study. Of note, such a high number remains feasible with LLMs: the cost of analyzing an abstract with GPT-4o-mini was about $0.00051 (March 2025).

While our results suggest that LLMs can be used as a potential alternative to search strategies for identifying RCTs, this was a relatively simple task. Further research is needed to evaluate their performance in more complex tasks such as identifying studies that meet specific content-related inclusion criteria. Such an approach would be particularly relevant in emerging research domains where terminology is evolving and where a single concept may be described using multiple synonyms or context-dependent phrases (e.g., in a review on just-in-time interventions, we used a very broad search strategy due to the absence of standardized terminology across disciplines). However, as shown in this study, leaving traditional search strategies could lead to a very high number of articles to screen (e.g., there were 179,000 records indexed in PubMed over 1 month). A potential solution could be to use a hybrid approach with a search limited to specific keywords to delineate the topic (e.g., disease of interest), followed by LLM-based assessment of the retrieved articles. Nevertheless, these approaches should be evaluated before being implemented.

## 6 Conclusion

Unlike Boolean queries relying on exact keyword matches, LLMs could therefore identify relevant studies based on meaning and semantic content rather than on form and words used in the title and abstract.

## Supporting information

Tran et al. supplementary material Tran et al. supplementary material

## Data Availability

The data that support the findings of this study are publicly available (abstract articles from electronic databases). A copy of the exact databases used in the study is provided in https://doi.org/10.5281/zenodo.16758565. The core functions required to process the data are appended to this manuscript.

## Supplementary material

To view supplementary material for this article, please visit http://doi.org/10.1017/rsm.2025.10034.
