# Supplementary material for: Using large language models to directly screen electronic databases as an alternative to traditional search strategies such as the Cochrane highly sensitive search for filtering randomized controlled trials in systematic reviews
Source: Res Synth Methods. 2025 Oct 10;16(6):1035–41. doi: 10.1017/rsm.2025.10034 (PMC12657644; doi:10.1017/rsm.2025.10034)
Supplement: Tran et al. supplementary material [file S1759287925100343sup001.docx]

**Supplementary materials**

# Search strategy

| **Initial search**  ("2024/09/01"[Date - Publication] : "2024/09/30"[Date - Publication]) AND "diabetes" AND (excludepreprints[Filter])  **Cochrane Highly sensitive search for RCTs**  ("2024/09/01"[Date - Publication] : "2024/09/30"[Date - Publication]) AND "diabetes" AND (excludepreprints[Filter]) AND (randomized controlled trial [pt] OR controlled clinical trial [pt] OR randomized [tiab] OR placebo [tiab] OR drug therapy [sh] OR randomly [tiab] OR trial [tiab] OR groups [tiab]) AND (excludepreprints[Filter]) AND (excludepreprints[Filter]) |
| --- |

# R code used to run the prompts through the API of GPT

The prompt uses the chat function of GPT models. For each abstract, it initiates a discussion with the first prompt, gets GPT answers and leverages it to further the discussion until the LLM provides a final decision.

| send_gpt_request <- function(prompt, api_key, model_name, chat_history) {  url <- "https://api.openai.com/v1/chat/completions"    if (is.null(chat_history)) {  chat_history <- data.frame(role = character(), content = character(), stringsAsFactors = FALSE)  }    new_entry <- data.frame(role = "user", content = prompt, stringsAsFactors = FALSE)  chat_history <- rbind(chat_history, new_entry)  messages <- lapply(seq_len(nrow(chat_history)), function(i) {  list("role" = chat_history[i, "role"], "content" = chat_history[i, "content"])  })    body <- toJSON(list(model = model_name, messages = messages), auto_unbox = TRUE)  headers <- c(  'Authorization' = paste('Bearer', api_key),  'Content-Type' = 'application/json'  )    response <- POST(url, body = body, encode = "json", add_headers(.headers = headers))  if (status_code(response) != 200) {  stop("Error in API request: ", content(response, "text"))  }    response_content <- fromJSON(content(response, "text", as = "text",encoding = "UTF-8"), flatten = TRUE)  ai_response <- response_content$choices$message.content  new_ai_entry <- data.frame(role = "system", content = ai_response, stringsAsFactors = FALSE)  chat_history <- rbind(chat_history, new_ai_entry)  return(list(response = ai_response, chat_history = chat_history))  }  prompter<-function(index, list)  {  chat_history <- data.frame(role = character(), content = character(), stringsAsFactors = FALSE)  prompt0 <- paste("You are an experienced systematic reviewer. You are screening medical research articles based on their title and abstracts to identify reports of randomized controlled trials. Appraise the following title and abstract. Summarize the elements that let you think that this study is (or is not) the report of a randomized controlled trial (including cross over, factorial trials, etc.). You must exclude secondary analyses, systematic reviews, meta-analyses, pooled analyses and protocols of randomized controlled trials, ### ### TITLE ###", listtit[index],paste("### ABSTRACT ###",listabs[index]))  response_and_history <- send_gpt_request(prompt0, api_key, model_name, chat_history)  chat_history <- response_and_history$chat_history  prompt1 <- paste("Based on the title and abstract and your previous analysis, should the record go to full-text screening (the record should go to full text screening if it is report of a randomized controlled trial or if there are not enough elements to decide whether it is one or not) ? This is less problematic to move texts to full-text screening than miss a potential randomized controlled trial")  response_and_history <- send_gpt_request(prompt1, api_key, model_name, chat_history)  chat_history <- response_and_history$chat_history  response_and_history <- send_gpt_request("Can you summarize your previous answer with simply \"yes\" or \"no\" ?", api_key, model_name, chat_history)  return(paste(response_and_history$chat_history$content))  } |
| --- |
